# A prospective observational study of the relationship of critical illness associated hyperglycaemia in medical ICU patients and subsequent development of type 2 diabetes

**DOI:** 10.1186/cc9101

**Published:** 2010-07-08

**Authors:** Ivan Gornik, Ana Vujaklija-Brajković, Ivana Pavlić Renar, Vladimir Gašparović

**Affiliations:** 1 Department of Intensive Care Medicine, University Hospital Centre Rebro, Kispaticeva 12, Zagreb 10000, Croatia; 2 Division of endocrinology, Department of Medicine, University Hospital Centre Rebro, Kispaticeva 12, Zagreb 10000, Croatia

## Abstract

**Introduction:**

Critical illness is commonly complicated by hyperglycaemia caused by mediators of stress and inflammation. Severity of disease is the main risk factor for development of hyperglycaemia, but not all severely ill develop hyperglycemia and some do even in mild disease. We hypothesised that acute disease only exposes a latent disturbance of glucose metabolism which puts those patients at higher risk for developing diabetes.

**Methods:**

Medical patients with no history of impaired glucose metabolism or other endocrine disorder admitted to an intensive care unit between July 1998 and June 2004 were considered for inclusion. Glucose was measured at least two times a day, and patients were divided into the hyperglycaemia group (glucose ≥7.8 mmol/l) and normoglycaemia group. An oral glucose tolerance test was performed within six weeks after discharge to disclose patients with unknown diabetes or pre-diabetes who were excluded. Patients treated with corticosteroids and those terminally ill were also excluded from the follow-up which lasted for a minimum of five years with annual oral glucose tolerance tests.

**Results:**

A five-year follow-up was completed for 398 patients in the normoglycaemia group, of which 14 (3.5%) developed type 2 diabetes. In the hyperglycaemia group 193 patients finished follow-up and 33 (17.1%) developed type 2 diabetes. The relative risk for type 2 diabetes during five years after the acute illness was 5.6 (95% confidence interval (CI) 3.1 to 10.2).

**Conclusions:**

Patients with hyperglycaemia during acute illness who are not diagnosed with diabetes before or during the hospitalization should be considered a population at increased risk for developing diabetes. They should, therefore, be followed-up, in order to be timely diagnosed and treated.

## Introduction

Hyperglycaemia commonly occurs in the course of any critical illness. This now generally known fact, first described by Claude Bernard in 1878 [1], became widely accepted after studies had shown its association with worse outcomes [2,3] and the positive effects of tight glucose control in the critically ill [4,5]. The issue is still focussed on after later studies [6] opened up a debate on how *tight *the control of glycaemia should be [7,8]. The usual idioms used for this phenomenon are *stress hyperglycaemia *and *critical illness hyperglycaemia *which include hyperglycaemia that occurs in patients with and without diabetes. The term *hospital acquired hyperglycaemia *[9] is proposed for hyperglycaemia in patients to whom no disorder of glucose metabolism can be diagnosed after the acute illness subsided.

The increase in blood glucose during acute illness is a consequence of complex mechanisms that are a part of stress and inflammatory responses. Cortisol is the main mediator of stress response, but other stress hormones such as catecholamines, glucagon and growth hormone also have hyperglycaemic effects [10,11]. Mediators of systemic inflammatory response, such as interleukin-1 (IL-1) and tumor necrosis factor alpha (TNF-α), cause hyperglycaemia and peripheral insulin resistance by inducing the release of *stress *hormones. They also alter insulin receptor signalling [12-16] and create insulin resistance. Due to these actions, glucose uptake in fat and muscle cells is reduced and hepatic gluconeogenesis is not suppressed despite hyperglycaemia. Consequent to inhibition of pancreatic beta-cells by cytokines and catecholamines, insulin concentrations may be normal or even decreased [17-19]. Medical interventions, such as enteral and parenteral nutrition, administration of vasopressors and glucocorticoids, add even further to disturbed glucose homeostasis. Despite the fact that endocrine and metabolic changes probably occur in all acutely ill patients, evident hyperglycaemia is not present in all of them. Its occurrence is certainly associated with the severity of illness, and has been associated with unfavourable outcomes in several acute conditions [2,3,20,21]. Nevertheless, all patients with severe infections, severe myocardial infarction or other critical illnesses do not develop hyperglycaemia and some will have hyperglycaemia even in milder disease. A patient's predisposition (pancreatic reserve and baseline insulin resistance) obviously plays an important part in the development of hyperglycaemia. We hypothesised that hospital acquired hyperglycaemia reveals this predisposition, that is, those patients are at risk for developing type 2 diabetes in the period subsequent to acute illness.

## Materials and methods

This was a prospective observational study performed in University Hospital Centre Rebro, Zagreb. Medical patients admitted to the intensive care unit during the period from July 1998 to June 2004 were included. Adult patients admitted to the ICU were evaluated for inclusion if they had a negative history of diabetes mellitus (DM), impaired fasting glucose (IFG), impaired glucose tolerance (IGT) or any other endocrine disorder. Patients receiving corticosteroid treatment and those with acute pancreatitis were not considered. For all other patients, blood glucose levels were measured at least twice a day (at 6 AM and 6 PM) during their ICU stay. The terms *fasting *and *postprandial *are intentionally omitted consequent to specific circumstances in critically ill patients. Additional glucose measurements were performed for patients with variable blood glucose or if insulin was administered for treatment of hyperglycaemia. Venous blood was analyzed on a point-of-care blood gas analyzer (IL GEM^® ^Premier™ 3000, Instrumentation Laboratories, Lexington, MA, USA). The threshold for hyperglycaemia was set at > 7.7 mmol/l (140 mg/dL), but all blood glucose measurements were recorded for analyses.

Patients were fed according to the Department policy. In short, all patients were fed from admission; all patients who could tolerate or had no counter indications were fed enterally (by mouth, gastric or jejunal tube); patients were fed parenterally if they did not tolerate enteral feeding; a combination of enteral and parenteral nutrition was given to patients who could not enterally receive targeted caloric intake set at 15 kCal/kg/day [22,23]. Mean achieved caloric intake (percent of target) was recorded for all patients.

To allow for better comparison of results, patients were divided into three groups according to their primary admission diagnosis: i) sepsis (including severe sepsis and septic shock); ii) acute coronary syndrome (myocardial infarction and unstable angina); and iii) all other admission diagnoses. This division was made due to the fact that sepsis and acute coronary syndromes combined account for more than two-thirds of medical ICU admissions in our hospital. Other admission diagnoses alone could not achieve a sufficient number of patients to be appropriately analysed separately.

Patients discharged from the hospital alive were asked to participate in the follow-up. Those who consented were tested using oral glucose tolerance test (OGTT) four to six weeks after discharge to exclude pre-existing (not diagnosed) impairment of glucose metabolism. Patients who were diagnosed with IGF, IGT or DM were excluded from follow-up. We also excluded patients with disseminated malignant disease, end-stage chronic disease or any other acute or chronic condition that was expected to cause early fatality and hinder planned five-year follow-up. At the beginning of the follow-up we recorded the patient's height and weight, cholesterol and triglyceride concentrations. All patients were given advice on positive lifestyle changes: dietary improvements, weight loss for the overweight, regular aerobic cardiovascular exercise, and so on.

During the follow-up period, all patients were contacted annually and their glycaemic status was evaluated by OGTT. If the diagnosis of DM, IFG or IGT was established independently from the study, amid yearly re-evaluations, the diagnosis was recorded without further confirmation. Patients diagnosed with DM, IFG or IGT were referred to an endocrinologist and were not followed up further. If a patient was diagnosed with another endocrine disorder or started receiving corticosteroids during the follow-up, he/she was excluded from the study. The follow-up was planned to last for at least five years, but yearly assessments were continued even longer when possible. We concluded the follow up on 31 July 2009.

### Definitions

Impaired fasting glucose (IFG), impaired glucose tolerance (IGT) and diabetes mellitus (DM) were defined according to the ADA criteria [24]. Sepsis, severe sepsis and septic shock were defined according to the usual criteria [25]. Acute coronary syndrome, unstable angina and myocardial infarction were defined according to the ACC/AHA criteria [26,27]

### Statistical analyses

MedCalc™ v. 9.6.2.0 (MedCalc Software, Mariakerke, Belgium). statistical software was used for all statistical analyses. Categorical data are presented as absolute and relative frequencies, continuous variables as median with inter-quartile range (IQR). Since distribution of data of the continuous variables did not always follow normal distribution, Wilcoxon's test was chosen for group comparisons of continuous variables. Chi square test was used for categorical variables. Statistical significance was set at α = 0.05.

The study was approved by the Ethics Committee of the University Hospital Centre. All patients included in the study signed an informed consent form. The study was not funded or supported by any organization, group or individual.

## Results

During the six inclusion years there were 2,207 ICU admissions, 1,822 with no history of hyperglycaemia or diabetes prior to the admission. Of those, 1,548 (90.6%) were discharged from the hospital alive and were considered for inclusion in the study. We excluded 211 patients who refused to participate in the study, 203 patients due to terminal illness, and another 29 patients who were receiving corticosteroid treatment.

Of the remaining 1,105 patients, 669 were normoglycaemic during the whole ICU stay and 436 had hyperglycaemia (venous blood glucose > 7.7 mmol/l). Diabetes or impaired glucose metabolism was diagnosed after discharge in 76 patients in the hyperglycaemia group which led to their exclusion from the follow-up decreasing hyperglycaemia group to 360 patients. The follow-up was thus initiated for 1,029 patients; their characteristics at baseline are given in Table 1. There were no differences in age and sex distribution. Patients in the hyperglycaemia group had a higher proportion of positive family history of diabetes and higher body mass indexes.

**Table 1:** Characteristics of patients in normoglycaemia and hyperglycaemia group at initiation of follow-up

	All patients (N = 1,029)	Patients with hyperglycaemia (N = 360)	Patients without hyperglycaemia (N = 669)	Hyperglycaemia vs. normoglycaemia
Diagnoses (N, %)				
- sepsis^a^	376	164 (43.6%)	202 (56.4%)	*P *< 0.001
- ACS^b^	322	97 (30.1%)	225 (69.9%)	
- other diagnoses	331	99 (29.9%)	232 (70.1%)	
Age (years)	58 (19 to 87)	59 (22 to 87)	58 (19 to 86)	*P *= 0.214
Male sex (N, %)	570 (55.4%)	194 (53.9%)	376 (56.2%)	*P *= 0.781
Body mass index (kg/m^2^)	27.3 (17.5 to 39.8)	29.4 (17.5 to 39.8)	26.8 (17.6 to 38.5)	*P *= 0.025
Family history of diabetes	108 (10.5%)	48 (13.3%)	60 (8.9%)	*P *= 0.038
Triglycerides (mmol/l)	1.4 (0.9 to 4.5)	1.4 (0.9 to 4.2)	1.3 (0.9 to 4.5)	*P *= 0.106
Cholesterol (umol/l)	4.5 (2.1 to 7.7)	4.8 (2.0 to 9.7)	4.9 (2.1 to 8.0)	*P *= 0.146
Glucose levels^c^	6.4 (2.7 to 23.5)	7.6 (3.8 to 23.5)	5.2 (2.7 to 7.7)	*P *< 0.001
				
Feeding regimen (N, %)				
- enteral nutrition only	703 (68.3%)	248 (68.8%)	455 (68.1%)	*P *= 0.823
- total parenteral or combination	326 (31.7%)	112 (31.1%)	214 (31.9%)	
Caloric intake (% of target)	85% (66 to 115)	88% (69 to 112)	84% (67 to 113)	*P *= 0.541

^a ^includes severe sepsis and septic shock

^b ^ACS, acute coronary syndrome (unstable angina and myocardial infarction)

^c ^Medians and ranges of all measured blood glucose levels for all patients in a group

Categorical data are presented as absolute and relative frequencies, continuous variables with medians with interquartile range.

**Figure 1:**
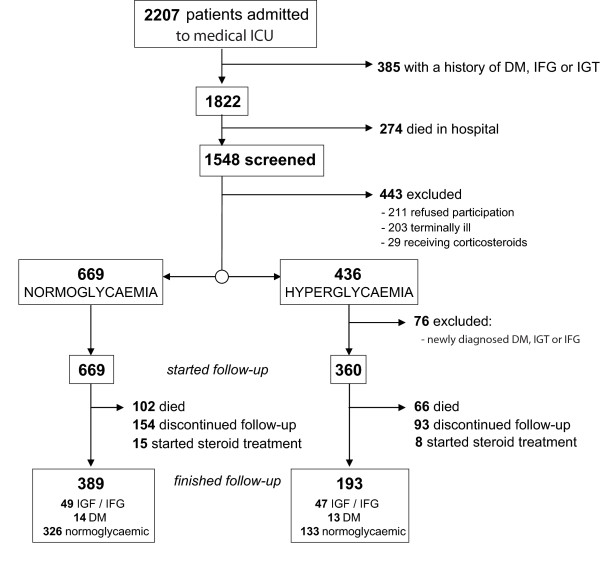
**Flow diagram showing the loss of patients from initial screening to the end of five-year follow-up**.

During the five years of follow-up, 102 (15.2%) patients in the normoglycaemia group and 66 (18.3%) patients in the hyperglycaemia group died. There were 154 patients in the normoglycaemia group and 93 in the hyperglycaemia group who discontinued their assessments. Also, we stopped the follow-up for 15 patients in the normoglycaemia group and 8 in the hyperglycaemia group because steroid treatment was initiated for treatment of various conditions. Figure 1 shows the flow diagram illustrating the patient disposition during follow-up.

Planned follow-up of five years was concluded for 591 patients. At the end of the follow-up there was no difference between the normoglycaemia and hyperglycaemia group in body mass index (25.2 (17.0 to 37.8) vs. 26.9 (18.1 to 39.4) respectively; *P *= 0.261). Loss of patients during the follow-up did not significantly affect other patients' characteristics from those at baseline (data not shown). The five-year follow-up was completed for 193 patients in the hyperglycaemia group of which 47 (24.4%) developed fasting hyperglycaemia or impaired glucose tolerance, while 33 (16.6%) developed type 2 diabetes. Of 398 patients in the normoglycaemia group 49 (12.3%) developed IFG or IGT, while 14 (3.5%) were diagnosed with type 2 diabetes mellitus during five years (Table 2). Chi-square test showed this to be a statistically significant difference (*P *< 0.001). According to these results, patients with hyperglycaemia (defined as glucose ≥7.8 mmol/l) during acute illness had a relative risk for developing type 2 diabetes of 5.6 (95% CI 3.1 to 10.2) and for developing IFG or IGT of 2.3 (95% CI 1.6 to 3.4).

**Table 2:** Incidence of impaired fasting glucose (IFG), impaired glucose tolerance (IGT) and type 2 diabetes mellitus (DM) during the five years follow-up after hospitalisation

	Hyperglycaemia group	Normoglycaemia group	Relative risk
**Finished follow-up**			
- sepsis^a^	70	139	
- ACS^b^	75	153	
- other diagnoses	48	106	
**all patients**	**193**	**398**	
**New IFG or IGT**			
- sepsis^a^	18	18	2.1 (95% CI 1.3 to 4.1)
- ACS^b^	19	17	2.6 (95% CI 1.4 to 4.6)
- other diagnoses	10	14	1.9 (95% CI 0.9 to 3.9)
**all patients**	**47 (24.4%)**	**49 (12.3%)**	**2.3 (95% CI 1.6 to 3.4)**
**New Type 2 DM**			
- sepsis^a^	13	6	5.0 (95% CI 2.0 to 12.5)
- ACS^b^	10	4	6.0 (95% CI 1.9 to 18.5)
- other diagnoses	10	4	6.0 (95% CI 2.0 to 18.1)
**all patients**	**33 (17.1%)**	**14 (3.5%)**	**5.6 (95% CI 3.1 to 10.2)**
**Remained normoglycaemic**			
- sepsis^a^	39	115	
- ACS^b^	46	132	
- other diagnoses	28	88	
**all patients**	**113 (58.5%)**	**335 (84.2%)**	

^a ^includes severe sepsis and septic shock

^b ^ACS, acute coronary syndrome (unstable angina and myocardial infarction

**Figure 2:**
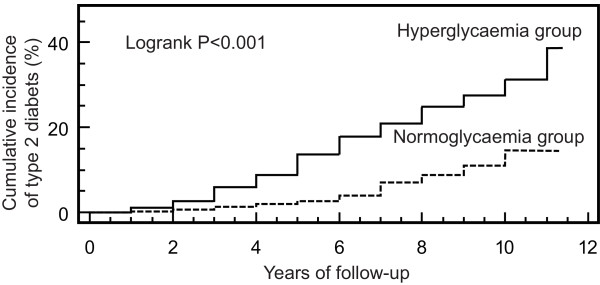
**Cumulative incidence of diabetes in patients with hyperglycaemia and normoglycaemia during critical illness**.

Patients included in the early years of the study were followed after the targeted five-year period; maximal follow-up time was 11 years for patients included in the first year. Cumulative incidence of diabetes during those 11 years is shown in Figure 2; Logrank analysis of the curves gives significant difference (*P *< 0.001). When we evaluated the three groups of diagnoses separately, we found that the absolute and relative risks for the onset of newly diagnosed impaired glucose metabolism were similar (Table 2).

## Discussion

Our results all point to an increased risk of developing diabetes mellitus or impaired glucose metabolism in the period following acute illness complicated with hyperglycaemia. There was no *tight *glucose control policy in our department during the inclusion years. Therefore, the glucose values measured are mostly *natural *levels, without intervention. Feeding regimen and caloric intake can play a role in development of hyperglycaemia, but they were not different between the groups. Most of the patients in both groups were enterally fed, and there was no difference in given caloric intake.

The patients with hyperglycaemia had a higher proportion of positive family history of diabetes and higher median body mass index which shows that usual risk factors for diabetes contribute to development of hyperglycaemia in acute illness. Although we cannot claim that those statistically significant differences have clinical relevance, they offer at least partial explanation for the increased risk of diabetes during follow-up. Whatever the underlying physiology, there is a combination of physiological factors predisposing a patient for hyperglycaemia in acute illness, during which hyperglycaemic mechanisms in stress and inflammatory response reveal the disorder. After the acute illness subsides, blood glucose returns to normal, but the disorder that led to hospital acquired hyperglycaemia remains and in some patients leads to overt impairment of glucose metabolism.

Metabolic disorders that make a patient prone to hyperglycaemia are a subject of speculation, but almost certainly include pre-existing increased insulin resistance and dysfunction of beta cells. Insulin resistance is present in the acutely ill [13,15,18,28] in different intensity, but the factors determining the extent of insulin resistance are not known. Our observation that body mass index, which is certainly associated with insulin resistance [29], is higher in the hyperglycaemia group offers part of the answer. Beta cell dysfunction was associated with respiratory and cardiac failure in critically ill children [30]. There are possibly some more disorders responsible for *tendency to hyperglycaemia *that are the root of hospital acquired hyperglycaemia and in the long term lead to development of diabetes.

Although the incidence of hospital acquired hyperglycaemia differed between the three subgroups of patients, the risk for diabetes is similar. The mechanisms contributing to hyperglycaemia differ among syndromes, especially between acute coronary syndromes, where inflammation probably plays a minor role and sepsis where systemic inflammation is an important contributing factor. The difference in the incidence of hyperglycaemia is probably a consequence of those differences and the differences in the severity of disease. However, it seems that it is not important what tilts the glycaemic control out of balance, since patients suffer comparable risks for development of DM, IFG or IGT.

This study was limited to medical ICU patients and its results may not apply to surgical patients, although the mechanisms leading to hyperglycaemia should be the same. A similar study on surgical populations is needed, until then we can only assume a similar effect. It is possible that surgical patients will need a higher cut-off for hyperglycaemia since hyperglycaemia is more common.

The definition of hospital acquired hyperglycaemia is not universal [31]. For instance, some studies used the same threshold that we did [32,33], one study compared three groups: glucose < 7.8 vs. 7.8 to 11.1 vs. glucose ≥11.1 mmol/l [34]. Other studies compared tertiles or sextiles of glycaemia [31]. We defined hospital acquired hyperglycaemia as glucose > 7.7 mmol/l (140 mg/dL), which is the cut-off value in the Recommendations of the American Heart Association [35] and the trigger for initiation of insulin treatment for ICU patients recommended by the American College of Endocrinology [36-38]. A higher threshold would probably reduce the hyperglycaemia group, but not necessarily increase the relative risk for diabetes, since it would put more patients with the presumptive disorder in the normoglycaemia group.

According to the literature, the incidence of hyperglycaemia ranges from about 30% to as high as 100% [30,39-44], depending on the severity of disease, patient case-mix and even more importantly on the chosen threshold for hyperglycaemia. Overall, our incidence of hyperglycaemia is similar to results published in the literature. Our case-mix had a high proportion of patients with ACS and sepsis. This can, in part, be explained by the fact that there are specialised intensive care units in the hospital that admitted specific diagnoses. ACS patients were admitted in high proportion because of the small number of beds available on cardiology wards and in the coronary care unit during the inclusion years.

We used OGTT during the follow-up for diagnosing DM, IGT and IFG. Glycated haemoglobin (HbA1c) was proposed [45] and has recently been recommended as a diagnostic test for diabetes and prediabetes [46]. We, however, did not measure HbA1c during hospitalisation or during follow-up since it was not officially recommended. However, HbA1c seems to be the optimal method for screening those patients in the future.

There appears to be a similarity between hyperglycaemia of critical illness and gestational diabetes [47]. Gestational diabetes, similar to hospital acquired hyperglycaemia, is a temporary disorder of glucose homeostasis, caused by failure of beta-cells to overcome insulin resistance created by the anti-insulin hormones secreted by the placenta [48]. The same risk factors predict GD and subsequent diabetes in women with a history of GD [49,50]. It is now generally recommended that women with GD should be screened regularly and should undergo secondary prevention to reduce and delay incidence of type 2 diabetes [51]. In a recent paper [52], 20 cohort studies of GD that included control group were identified; cumulative relative risk for type 2 DM was 7.43 (95% CI 4.79 to 11.51).

The size of our study remains its strongest limitation and we are now planning a large, multi-centre study to further substantiate our results. The majority of studies on gestational diabetes [52] are, however, comparable to ours or even smaller in the number of patients included and follow-up time.

Current prevalence of hyperglycaemic conditions (over 40% of adult Americans [53]) has reached epidemic proportions. Recognising conditions that unveil the risk of developing diabetes and prediabetes is thus of great practical value.

Our present results suggest that the patients with hyperglycaemia during acute illness, who are not diagnosed with pre-diabetes or diabetes during or immediately after hospitalisation, should be perceived as patients with increased risk of developing diabetes and should as such be regularly monitored and treated appropriately. According to the recent recommendations [46], annual HbA1c measurements could be used for monitoring such patients.

## Conclusions

Hyperglycaemia occurring during critical illness in non-diabetic medical patients is associated with increased risk of developing diabetes in the five-year period after the discharge. Stress and inflammation during acute illness seem to reveal an inherent disorder of glucose metabolism which in the following years leads to development of diabetes.

## Key messages

• Non-diabetic patients with hyperglycaemia (> 7.7 mmol/l) during critical illness are at increased risk of developing type 2 diabetes or glucose intolerance in the period following recovery

• Patients with hyperglycaemia in whom pre-existing diabetes is excluded after the recovery of acute illness should be followed-up to diagnose the occurrence of overt disorders of glucose metabolism and to timely start treatment

## Abbreviations

ADA: American Diabetes Association; DM: diabetes mellitus; GD: gestational diabetes; IFG: impaired fasting glucose; IGT: impaired glucose tolerance; IL-1: interleukin 1; IQR: inter-quartile range; OGTT: oral glucose tolerance test; TNF-α: tumor necrosis factor alpha.

## Competing interests

The authors declare that they have no competing interests.

## Authors' contributions

IG conceived and organized the study, participated in patients' inclusion and follow-up, performed statistical analyses, analyzed results and wrote the manuscript. AVB and VG participated in patients' inclusion and follow-up. IPR was involved in the analysis of results and writing the manuscript. All the authors read and approved the final version.
